# Mapping of the Micro-Mechanical Properties of Human Root Dentin by Means of Microindentation

**DOI:** 10.3390/ma14030505

**Published:** 2021-01-21

**Authors:** Michael Kucher, Martin Dannemann, Niels Modler, Martina Romy Bernhard, Christian Hannig, Marie-Theres Weber

**Affiliations:** 1 Institute of Lightweight Engineering and Polymer Technology (ILK), Technische Universität Dresden, Holbeinstraße 3, 01307 Dresden, Germany; michael.kucher@tu-dresden.de (M.K.); niels.modler@tu-dresden.de (N.M.); 2 Clinic of Operative and Pediatric Dentistry, Medical Faculty Carl Gustav Carus, Technische Universität Dresden, Fetscherstraße 74, 01307 Dresden, Germany; romy.bernhard@mailbox.tu-dresden.de (M.R.B.); christian.hannig@uniklinikum-dresden.de (C.H.); marie-theres.weber@uniklinikum-dresden.de (M.-T.W.)

**Keywords:** creep, human root dentin, indentation elastic modulus, mapping, Martens hardness, microindentation, Vickers hardness

## Abstract

The extensive knowledge of root dentin’s mechanical properties is necessary for the prediction of microstructural alterations and the teeth’s deformations as well as their fracture behavior. Standardized microindentation tests were applied to apical, medial, and cervical root sections of a mandibular human first molar to determine the spatial distribution of the hard tissue’s properties (indentation modulus, indentation hardness, Martens hardness, indentation creep). Using an indentation mapping approach, the inhomogeneity of mechanical properties in longitudinal as well as in transversal directions were measured. As a result, the tooth showed strongly inhomogeneous material properties, which depended on the longitudinal and transversal positions. In the transversal cutting planes of the cervical, medial, apical sections, the properties showed a comparable distribution. A statistical evaluation revealed an indentation modulus between 12.2 GPa and 17.8 GPa, indentation hardness between 0.4 GPa and 0.64 GPa and an indentation creep between 8.6% and 10.7%. The established standardized method is a starting point for further investigations concerning the intensive description of the inhomogeneous mechanical properties of human dentin and other types of dentin.

## 1. Introduction

A human tooth consists mainly of dentin. In dentistry, extensive knowledge of the mechanical properties of dentin microstructure regarding dentin alterations (abrasive and erosive processes, sclerotic dentine) and the fracture behavior, as well as the interaction with bonding agents and restorative materials, is required. Kinney et al. summarized the reliable ranges for the magnitudes of mechanical properties of human dentin, such as hardness, strength, fracture toughness, and fatigue, in a literature review [1]. A further aim of the experimental characterization is the prediction of the deformations of human dentin under different applied loads and under the influence of different surrounding conditions. For the modelling of the entire tooth, measurements of the mechanical properties are required. Grzebieluch et al. [2] modelled the mechanical properties of human dentin based on the concept of representative volume element (RVE) using the finite element method. The authors used the elastic properties determined by Kinney et al. [3] for the RVE modelling of dentin with a certain tubule orientation.

Several authors investigated the mechanical properties of dentin by means of compression testing [4], four-point bending flexural testing [5,6], microhardness testing [7,8,9,10,11] or nanoindentation [12,13,14]. Cuy et al. [15] mapped out the properties of enamel over the transversal cross-section of a maxillary molars, whereas Wang et al. [14] investigated the dentin-enamel junction at different locations by means of nanoindentation. For human root dentin, no investigation exists concerning the whole distribution of the mechanical properties in the longitudinal and transversal direction of the teeth.

The aim of the current study was to demonstrate a standardized measurement method determining the distribution of the mechanical properties (indentation modulus, Martens hardness, indentation hardness, indentation creep) for human root dentin for the first time. Therefore, the root of a tooth was sectioned in longitudinal and transversal segments, and the micro-mechanical properties of the cutting surfaces were measured by means of microindentation.

## 2. Materials and Methods

### 2.1. Sample Preparation

For the current investigation, a mandibular first molar was exemplary selected to show the applicability of the demonstrated measurement method. The tooth was collected from the Clinic of Oral and Maxillofacial surgery. It was extracted for medically justifiable reasons without any connection to the present study. No information was available about the patients’ name, age, sex, or general health condition. The tooth was not endodontically treated, and tooth’s root was not fractured and had no carious lesions. The molar was stored in 0.1% thymol solution at 6 °C for 9 weeks. The tooth was cleaned from calculus as well as from hard and soft tissue previously to examination.

**Figure 1:**
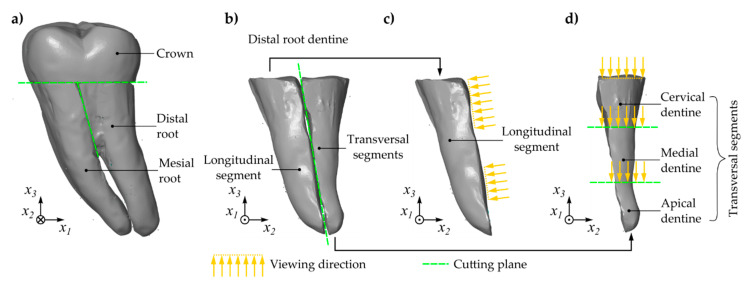
Sample preparation of human molar: separation of crown (lingual view), (**a**); partition of root dentin (distal view), (**b**); longitudinal segment (distal view), (**c**); partition of distal half (distal view), (**d**).

The tooth root was sectioned by means of a precision cutting machine with permanent water cooling (Accutom-50, Struers GmbH, Willich, Germany). The tooth crown was removed at the level of the cemento-enamel junction. The mesial and the distal root were separated from each other (Figure 1a). The distal root was further divided into two parts along the longitudinal axis in the mesio-distal direction. One of these parts represented the longitudinal segment (LS). The other part was further sectioned into three segments, in the following denoted as cervical segment (CS), medial segment (MS), and apical segment (AS).

Each segment was embedded in acrylic cold mounting resin for mounting. The blocks were then ground using a motorized polishing wheel (Rotopol-15, Struers GmbH, Willich, Germany) with interchangeable silicon carbide grinding discs of diminishing grit size (800 grit, 1200 grit, 2400 grit, and 4000 grit). Between each grinding step and at the end of polishing, the sample surfaces were gently cleansed of debris using running water and the cutting surfaces were blown off with air. To visualize the tooth anatomy, images of each cutting surface were taken by means of a stereo microscope (Zeiss Stemi 2000-C ZOOM, Carl Zeiss Jena, Jena, Germany) with an attachment system 0.3× and viewing magnification of 1.

### 2.2. Microindentation Measurements

**Figure 2:**
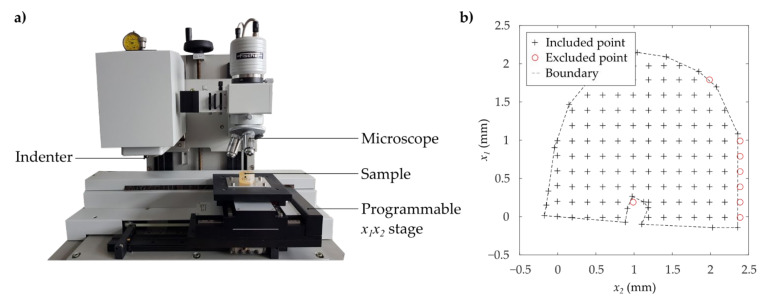
(**a**) Experimental setup and (**b**) example of the applied measuring grid of microindentation testing.

The mechanical properties of the different segments were analyzed using instrumented microindentation tests following VDI/VDE 2616 [16] (Figure 2). By means of an automated microindentation measuring system with programmable xy-table (Fischerscope H 100 C, Helmut Fischer GmbH, Sindelfingen, Germany), the mechanical properties were determined applying a measuring grid with a distance between points of 0.2 mm in $x_{1}$-direction and 0.2 mm in $x_{2}$-direction for the transversal segments and 0.3 mm in $x_{1}$-direction and 0.3 mm in $x_{3}$-direction for the longitudinal segment, respectively. All measurements were carried out at standard atmosphere.

The microindentation tests were carried out using an ideal Vickers pyramid. Using the curves of the indentation depth h under test force F while the test force increased, the Martens hardness HM and indentation hardness HIT were determined as described by [16]:

(1) $$HM=\frac{F}{26.43h^{2}}$$

(2) $$HIT=\frac{F}{24.43h_{c}^{2}}$$

The indentation hardness HIT is related to the material’s resistance to permanent deformation and was calculated by means of the contact depth $h_{c}$. In contrast, the Martens hardness HM is related to both the plastic and the elastic material properties. The increase in the indentation depth starting from an indentation depth $h_{1}$ to an increased depth $h_{2}$ after the application of the maximum test force describes the material’s creeping. The indentation creep CIT was calculated as follows [17]:

(3) $$CIT=\frac{h_{2}-h_{1}}{h_{1}}100\%$$

The indentation modulus EIT describes the elasticity of the material. Using the tangent of the decrease curve, the calculation of the indentation modulus was described by [17]:

(4) $$\frac{EIT}{1-\nu_{s}^{2}}=\frac{1}{\frac{1}{E_{r,n}}-\frac{1-\nu_{i}^{2}}{E_{i}}}$$

Thereby, Poisson’s ratio of the specimen, $\nu_{s}$, Poisson’s ratio of the indenter $\nu_{i}$, the reduced modulus at contact $E_{r,n}$, and the elastic modulus of the indenter $E_{i}$ were considered.

**Figure 3:**
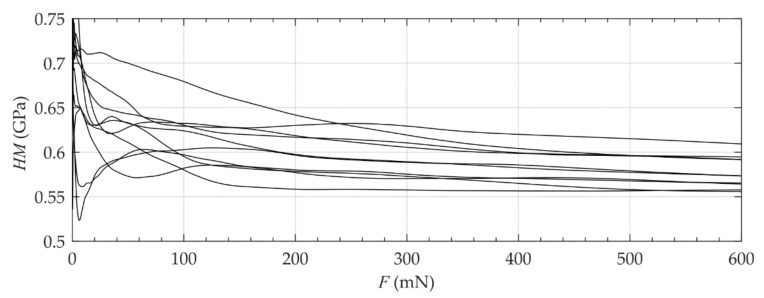
Influence of the maximum applied test force (10 measurements on the cutting surface of the tooth).

For the selection of a suitable test force, a comparable human molar was used. This tooth for preliminary investigations was prepared under equal conditions as the detailed investigated tooth of the current study. For this tooth, a variation of the test force F and the related Martens hardness HM showed a reliable reproducibility of the measured values for forces greater than 600 mN (compare Figure 3). This force ensured a sufficient maximum indentation depth with values between 5.8 µm and 11.2 µm. According to the requirement h≥20Ra of the indentation testing, the arithmetic average roughness Ra was measured by means of a three-dimensional measurement system (µscan, NanoFocus AG, Oberhausen, Germany) following DIN EN ISO 4287 [18]. Therefore, a representative quadratic area with a dimension of 1.3 mm of the cervical and the longitudinal segment was optically measured with an equidistant resolution of 1.5 µm. An averaged roughness of Ra = 0.1 ± 0.07 µm was measured for the cervical segment and an averaged roughness of Ra = 0.06 ± 0.006 µm was determined for the longitudinal segment. Using the maximum test force of 600 mN, the requirement of a sufficient indentation depth for the given surface roughness was fulfilled.

**Figure 4:**
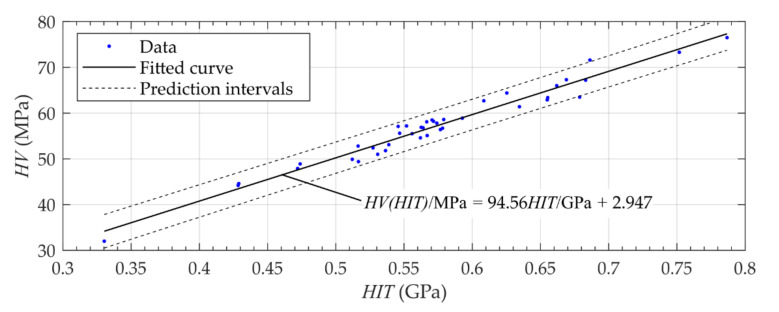
Functional relationship between indentation hardness HIT and the Vickers hardness HV with confidence bounds (confidence level of 0.95, resulting adjusted R-square of 0.96).

On the basis of a large number of comparative measurements, the values of HIT were converted into Vickers hardness HV. Therefore, a functional relationship between the optically measured Vickers hardness HV and the related indentation hardness HIT was determined by means of a linear least square fit. This yielded the following functional correlation (Figure 4):

(5) $$\frac{HV}{\mathrm{MPa}}\;=\;\frac{94.56HIT}{\mathrm{GPa}}+2.947$$

**Figure 5:**
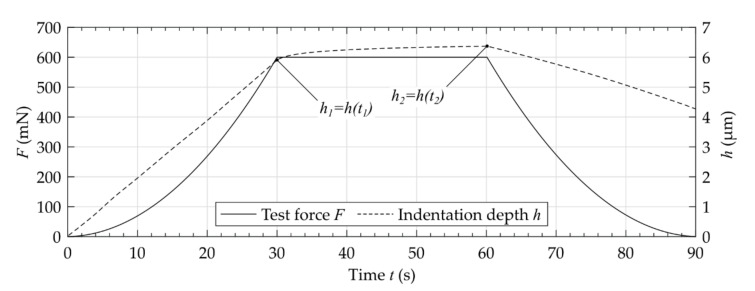
Exemplarily measured curve of test force and indentation depth.

According to the results of the preliminary investigations, all measurements were performed with a maximum test force $F_{\max}$ = 600 mN, an application time of the test force of $t_{F}$ = 30 s, a dwell time of the test force of $t_{d}$ = 30 s, and a time for removing the test force of $t_{r}$ = 30 s (compare Figure 5). The test force was applied with a constant gradient of $\partial \sqrt{F}/\partial t$. The measurements showed that the dentin was prone to creep after applying the maximum test force. After a period of 30 s, the creep rate decreased. The measurements of the properties HIT, HM, $EIT/\left(1-\nu_{s}^{2}\right)$ and CIT were performed and evaluated using the manufacturer’s original analyzing software (WIN-HCU 7.7, Helmut Fischer GmbH, Sindelfingen, Germany).

### 2.3. Spatial Analysis of Measured Data

The indentation measurements obtained the mechanical properties as a function of their current position located at the cutting surface of the different segments. To identify the anatomical boundary of the tooth, an additional measurement following the outer contour of the tooth was carried out (Figure 2). Only values within and on this boundary were included in the following investigations. Additionally to the investigation of mechanical properties maps, the mechanical properties at certain regions of interest (ROI) were analyzed. Therefore, lines were defined manually as ROI by means of an interactive draggable and resizable line. Therefore, the built-in function imline of the numerical computing environment (MATLAB 9.5, MathWorks, Inc., Natick, MA, USA) was used. For the LS, the mechanical properties related to lines approximately parallel to the longitudinal axis z starting from the apical dentin were determined. The transversal segments (CS, MS, AS) were analyzed using radial oriented lines, which were drawn through the approximated center of the root canal. For each line, the origin was located at the boundary of the root and the end was the outer contour of the root. The coordinate of length l of the lines in longitudinal and radial direction was normalized by their total length L. A two-sided Wilcoxon rank sum test with a significance level of α = 0.05 was carried out to show differences between the medians of the micro-mechanical properties evaluated for all values of measurement area and evaluated for the values defined by the certain lines.

## 3. Results

### 3.1. Mechanical Properties of Human Dentin

**Figure 6:**
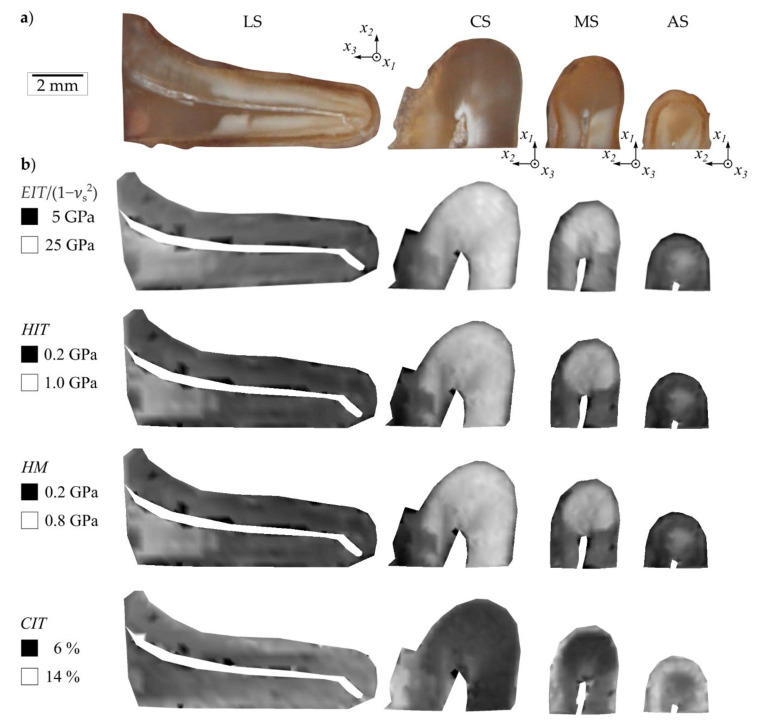
(**a**) Anatomy of mandibular first molar (longitudinal segment (LS), cervical segment (CS), medial segment (MS), apical segment (AS,) and (**b**) the related spatial distribution of mechanical properties: indentation modulus EIT, indentation hardness HIT, Martens hardness HM, indentation creep CIT.

**Figure 7:**
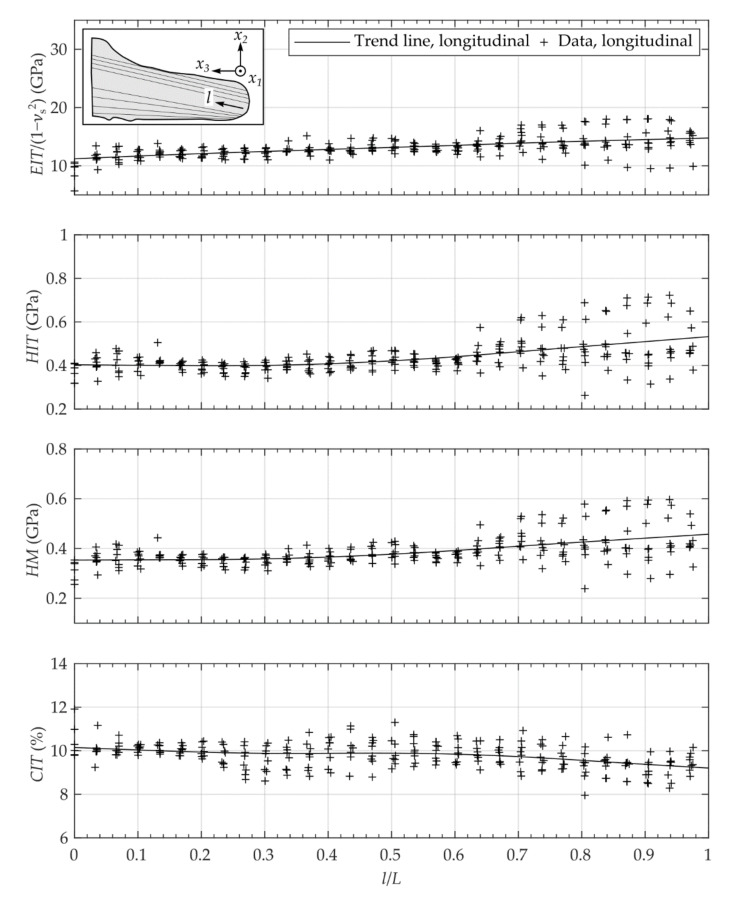
Micro-mechanical properties of the longitudinal segment evaluated at cutting lines (8 lines).

**Figure 8:**
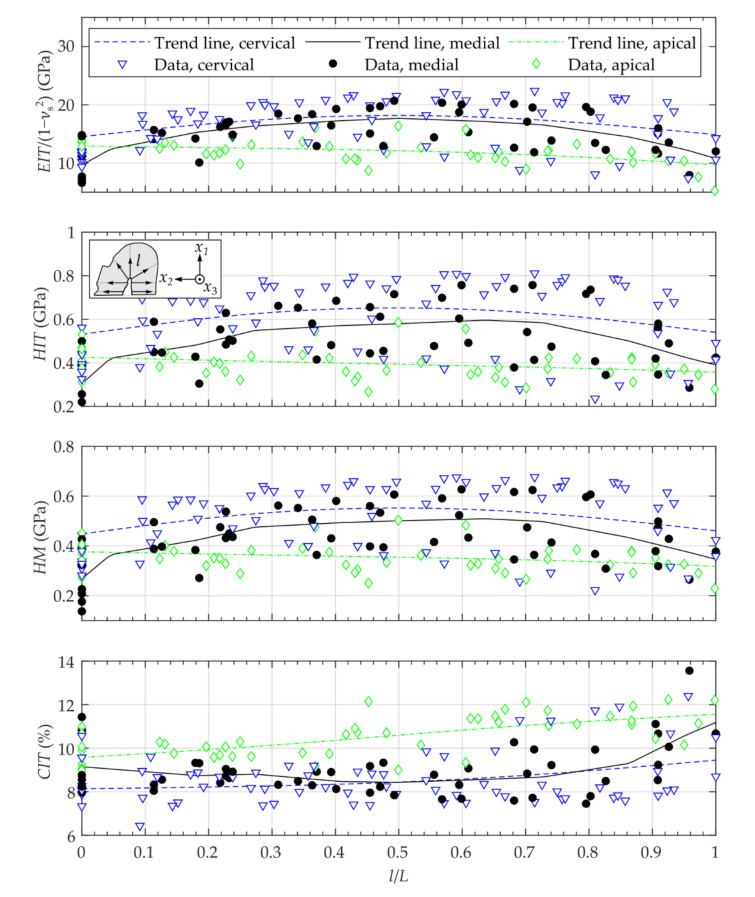
Micro-mechanical properties of the transversal segments evaluated at cutting lines (7 lines).

The measurements of the mechanical properties show an inhomogeneous distribution over the investigated cutting surfaces (Figure 6). Comparing the anatomy of the different segments, there was partially a relationship between dark-colored areas and the resulting properties. The measurements showed a viscoelastic-plastic deformation behavior during the applied indentation procedure (Figure 5). Comparing the transversal segments (CS, MS, AS), similar distributions of the mechanical properties are visible. As expected, the inhomogeneity of properties was also visible for the different lines in longitudinal as well as radial orientation (Figure 7 and Figure 8).

The statistical evaluation of the measured values of the entire area resulted partially in similar results as the values determined at the different ROIs (Table 1). Both statistical evaluations showed a high standard deviation which was related to the material’s inhomogeneity. As expected, the cervical dentin has the highest mean values of the mechanical properties $EIT/\left(1-\nu_{s}^{2}\right)$, HIT, and HM. The indentation creep obtained the lowest value for the cervical third.

### 3.2. Spatial Description of Mechanical Properties

The analysis of the different lines in longitudinal as well as radial orientation showed similar spatial courses (Figure 7 and Figure 8). For the longitudinal segment, the mean values of $EIT/\left(1-\nu_{s}^{2}\right)$, HIT and HM as well as the statistical scattering increased whereas the value of CIT slightly decreased with increasing distance from the apex (Figure 7). Analo­gously, the mechanical properties $EIT/\left(1-\nu_{s}^{2}\right)$, HIT and HM, of the apical segment showed lower values than the values of the cervical segment (Figure 8, Table 1).

In radial direction of the different transversal segments, the values of the mechanical properties $EIT/\left(1-\nu_{s}^{2}\right)$, HIT and HM had their maximum between l/L=0.5 and l/L=0.8 (Figure 8). The indentation creep showed the highest values for the apical segment and slightly decreased with increasing distance from the root canal center to the outer contour of the tooth (Figure 8).

## 4. Discussion

The findings of the current study show that human root dentin has a highly inhomogeneous viscoelastic-plastic deformation behavior. The investigated longitudinal segment showed an increase in the elastic modulus and the hardness as well as a decrease in the creep starting from the apical to the cervical region of the tooth. For most of the investigated transversal segments, the modulus and the hardness of the root dentin had lower values at the outer regions (root canal, periodont). Regarding the creep, the values were higher in the outer regions for most of the transversal segments. A radial symmetry of the spatial distribution of the micro-mechanical properties was visible. To identify these relationships, different linear evaluation paths were defined and statistically analyzed. The alignment as well as the location of the root canal sections was related to the tubule orientation of the root dentin (compare [6]). Therefore, center points were located within the main root canal being approximated at its center as demonstrated by Kucher et al. [19] for the transversal segments. Starting from this center point, approximately radially oriented measurements were performed. For the longitudinal segment and in the longitudinal direction, the orientation of the dentin tubules was similar.

Arola et al. [5] investigated the flexural modulus of beam (human dentin of different age groups) by means of four-point bending tests for different ages. They obtained values between 12 and 17 GPa; however, these values should be considered as integral values for the entire length of the specimen. Using the same experimental setup, Arola et al. [6] further analyzed the influence of the tubule orientation and obtained an averaged flexural modulus of 18.7 GPa for the beam length aligned parallel to tubules and 15.5 GPa perpendicular alignment, respectively. Fonseca et al. [8] characterized, among other things, the effect of Radiodensity on the Knoop hardness for one human third molar (enamel, dentin), but they analyzed only five indentations per specimen. He et al. [9] measured the Knoop hardness of dental hard tissue along specific lines at different distances from the outer enamel to the dentin-enamel junction. Liang et al. [13] measured among other things the nano-mechanical properties of dentin by means of nanoindentation with only six indentations per sample and obtained an indentation modulus of 29.3 GPa and a hardness of 0.78 GPa. Saghiri et al. [10] analyzed the relation between erosion and microhardness of root canal dentin by using a Vickers microhardness tester. The average of three indentations was calculated at two different distances from the pulp dentin interface. Hereby, values of HV 3 N/20 s = 40–47 N/mm^2^ (at 100 µm) and of HV 3 N/20 s = 50–57 N/mm^2^ (at 500 µm) were obtained. Wang et al. [14] investigated intensively the spatial distribution of the mechanical properties of dental hard tissue in the area of the dentin-enamel junction by means of nanoindentation and obtained an indentation modulus between 15 and 20 GPa and a hardness in the range from 0.7 to 0.8 GPa. In addition, the gradient of the mechanical properties at different intratooth locations crossing the dentin-enamel junction was evaluated. Zaytsev et al. [4] determined among other things the Young’s modulus of cuboids for human dentin under uniaxial compression, which resulted in a modulus of approximately 4 GPa. The measurement of certain areas of dentin was carried out by Kinney et al. [12], representing only a very small selection of measured data. The investigation of pre-defined regions such as demonstrated by Constantino et al. [20] obtained a better understanding of the spatial distribution, but these investigations were carried out for the enamel of primates. Cuy et al. [15] determined the nano-mechanical properties and distributions of enamel for longitudinal segments.

Compared to these previous studies, the application of the microindentation mapping enabled a detailed spatially resolved measurement of the micro-mechanical properties of dental hard tissue at different locations and in different orientations of the entire tooth. Furthermore, the demonstrated evaluation enabled the identification of patterns or symmetries in the spatial distribution of the mechanical properties. The introduced microindentation mapping approach was carried out for the investigation of the entire human root dentin for the first time. This represents an automatable and reproducible measurement method and at the same time has lower requirements for specimen preparation than nanoindentation. The mounting of the investigated specimen allowed the measurement of a high number of measurement points with minimal relocation. To compare the values of hardness measurements to previous studies (such as the study by Saghiri et al. [10]), the values of the indentation hardness were converted into Vickers hardness HV 0.6 N/30 s.

However, there were also some limitations of the introduced approach. In the boundary region (outer contour of the tooth), an influence of the measured values cannot be completely rejected since the resin does not have the same mechanical properties as the investigated dentin. A minimum distance between the measuring points is required to prevent the interactions between the individual measurements. This distance limits the maximum resolution of the measuring grid.

Nevertheless, the microindentation mapping of human root dentin enables an intensive characterization and evaluation of the inhomogeneous distributed micro-mechanical properties of dental hard tissue to identify patterns and symmetries. The introduced approach is a starting point for further investigations with higher numbers of specimens and/or pathologic altered dental hard tissues.

## 5. Conclusions

The standardized microindentation method enabled the mapping of the inhomogeneous viscoelastic-plastic material behavior of human root dentin. The evaluation of the spatially resolved micro-mechanical properties showed similar spatial distributions for the longitudinal segment in the longitudinal direction as well as for the transversal tooth segments in the radial direction. As a result, a punctual measurement of the micro-mechanical properties of dentin cannot be used for the assessment of the complete dental root.

## Figures and Tables

**Table 1:** Statistical evaluation of the mandibular molar (mean value ± standard deviation (probability value p)).

Value.	Longitudinal	Cervical	Medial	Apical
Evaluation of Entire Area				
$EIT/\left(1-\nu_{s}^{2}\right)$ in GPa	13.7 ± 2.34	17.8 ± 4.38	16 ± 3.09	12.2 ± 1.96
HIT in GPa	0.47 ± 0.102	0.64 ± 0.171	0.54 ± 0.133	0.4 ± 0.072
HV in MPa	47 ± 12.6	63.5 ± 19.13	53.7 ± 15.55	41.2 ± 9.78
HM in GPa	0.41 ± 0.081	0.54 ± 0.136	0.47 ± 0.101	0.36 ± 0.058
CIT in %	9.5 ± 0.82	8.6 ± 1.21	9.1 ± 1.3	10.7 ± 1.03
Evaluation of Lines				
$EIT/\left(1-\nu_{s}^{2}\right)$ in GPa	13.1 ± 1.72 (p < 0.01)	16.9 ±4.13 (p < 0.01)	15.3 ± 3.36 (p = 0.04)	11.8 ± 1.99 (p = 0.3)
HIT in GPa	0.43 ± 0.077 (p < 0.01)	0.61 ± 0.168 (p < 0.01)	0.51 ± 0.137 (p = 0.25)	0.39 ± 0.069 (p = 0.13)
HV in MPa	44.5 ± 10.2 (p < 0.01)	60.6 ± 18.79 (p < 0.01)	51.8 ± 15.92 (p = 0.25)	40.1 ± 9.51 (p = 0.13)
HM in GPa	0.38 ± 0.06 (p < 0.01)	0.52 ± 0.132 (p < 0.01)	0.45 ± 0.108 (p = 0.13)	0.35 ± 0.057 (p < 0.09)
CIT in %	9.8 ± 0.57 (p < 0.01)	8.6 ± 1.25 (p = 0.48)	9 ± 1.09 (p = 0.99)	10.6 ± 0.91 (p = 0.26)

## Data Availability

The data presented in the current study are available on request from the corresponding author.

## References

[1] Kinney J.H., Marshall S., Marshall G. (2003). The Mechanical Properties of Human Dentin: A Critical Review and Re-evaluation of the Dental Literature. Crit. Rev. Oral Biol. Med..

[2] Grzebieluch W., Romuald B., Tomasz C., Kaczmarek U. (2017). The mechanical properties of human dentin for 3-D finite element modeling—Numerical and analytical evaluation. Adv. Clin. Exp. Med..

[3] Kinney J., Balooch M., Marshall G., Marshall S. (1999). A micromechanics model of the elastic properties of human dentine. Arch. Oral Biol..

[4] Zaytsev D., Grigoriev S., Panfilov P. (2012). Deformation Behavior of Human Dentin under Uniaxial Compression. Int. J. Biomater..

[5] Arola D., Reprogel R. (2005). Effects of aging on the mechanical behavior of human dentin. Biomaterias.

[6] Arola D., Reprogel R.K. (2006). Tubule orientation and the fatigue strength of human dentin. Biomaterias.

[7] Craig R., Peyton F. (1958). The Microhardness of Enamel and Dentin. J. Dent. Res..

[8] Fonseca R.B., Haiter-Neto F., Carlo H., Soares C.J., Geraldeli S., Puppin-Rontani R.M., Correr-Sobrinho L. (2008). Radiodensity and hardness of enamel and dentin of human and bovine teeth, varying bovine teeth age. Arch. Oral Biol..

[9] He B., Huang S., Jing J., Hao Y. (2010). Measurement of hydroxyapatite density and Knoop hardness in sound human enamel and a correlational analysis between them. Arch. Oral Biol..

[10] Saghiri M.A., Delvarani A., Mehrvarzfar P., Malganji G., Lotfi M., Dadresanfar B., Saghiri A.M., Dadvand S. (2009). A study of the relation between erosion and microhardness of root canal dentin. Oral Surg. Oral Med. Oral Pathol. Oral Radiol. Endodontol..

[11] Xu H., Smith D., Jahanmir S., Romberg E., Kelly J., Thompson V., Rekow E. (1998). Indentation Damage and Mechanical Properties of Human Enamel and Dentin. J. Dent. Res..

[12] Kinney J., Balooch M., Marshall S., Marshall G., Weihs T. (1996). Hardness and young’s modulus of human peritubular and intertubular dentine. Arch. Oral Biol..

[13] Liang X., Zhang J.Y., Cheng I.K., Li J.Y. (2016). Effect of high energy X-ray irradiation on the nano-mechanical properties of human enamel and dentine. Braz. Oral Res..

[14] Wang Z., Wang K., Xu W., Gong X., Zhang F. (2018). Mapping the mechanical gradient of human dentin-enamel-junction at different intratooth locations. Dent. Mater..

[15] Cuy J., Mann A., Livi K., Teaford M., Weihs T.P. (2002). Nanoindentation mapping of the mechanical properties of human molar tooth enamel. Arch. Oral Biol..

[16] VDI/VDE 2616 Blatt 2 Verein Deutscher Ingenieure e.V (2014). Hardness Testing of Plastics and Elastomers.

[17] DIN EN ISO 14577-1 Deutsches Institut für Normung e.V (2015). Metallic Materials—Instrumented Indentation Test for Hardness and Materials Parameters—Part. 1: Test. Method.

[18] DIN EN ISO 4287 Deutsches Institut für Normung e.V (2010). Geometrical Product Specifications (GPS)—Surface Texture: Profile Method—Terms, Definitions and Surface Texture Parameters.

[19] Kucher M., Dannemann M., Modler N., Haim D., Hannig C., Weber M.-T. (2020). Continuous Measurement of Three-Dimensional Root Canal Curvature Using Cone-Beam Computed and Micro-Computed Tomography: A Comparative Study. Dent. J..

[20] Constantino P.J., Lee J.J.-W., Gerbig Y., Hartstone-Rose A., Talebi M., Lawn B.R., Lucas P.W. (2012). The role of tooth enamel mechanical properties in primate dietary adaptation. Am. J. Phys. Anthropol..

